# Facemask and Respirator in Reducing the Spread of Respiratory Viruses; a Systematic Review

**DOI:** 10.22037/aaem.v9i1.1286

**Published:** 2021-08-16

**Authors:** Negin Shaterian, Fatemeh Abdi, Zahra Atarodi Kashani, Negar Shaterian, Mohammad Darvishmotevalli

**Affiliations:** 1 Student Research Committee, School of Nursing & Midwifery, Shahid Beheshti University of Medical Sciences, Tehran, Iran.; 2 School of Nursing and Midwifery, Alborz University of Medical Sciences, Karaj, Iran.; 3 Non-Communicable Diseases Research Center, Alborz University of Medical Sciences, Karaj, Iran.; 4 Department Midwifery, Iranshahr University of medical sciences, Iranshahr, Iran.; 5 Student Research Committee, Jahrom University of Medical Sciences, Jahrom, Iran.; 6 Research Center for Health, Safety and Environment , Alborz University of Medical Sciences, Karaj, Iran.

**Keywords:** Masks, respiratory protective devices, respiratory tract infections, virus diseases, N95 respirators

## Abstract

**Introduction:**

Respiratory viruses spread fast, and some manners have been recommended for reducing the spread of these viruses, including the use of a facemask or respirator, maintaining hand hygiene, and perfoming social distancing. This systematic review aimed to assess the impact of facemasks and respirators on reducing the spread of respiratory viruses.

**Methods:**

We conducted a systematic review using MeSH terms, and reported findings according to PRISMA. PubMed, Embase, Cochrane Library, Scopus, ProQuest, Web of Science(WoS), and Google Scholar were searched for articles published between 2009 and 2020. Two independent reviewers determined whether the studies met inclusion criteria. The risk of bias of studies was assessed using Newcastle-Ottawa (NOS) and Consolidated Standards of Reporting Trials (CONSORT).

**Results:**

A total of 1505 articles were initially retrieved and 10 were finally included in our analysis (sample size: 3065). 96.8% of non-infected participants used facemask or respirator in contact with people infected with a respiratory virus, facemask and respirator have a significant effect on reducing the spread of respiratory viruses.

**Conclusion:**

Evidence support that using a facemask or respirator can reduce the spread of all types of respiratory viruses; therefore, this result can be generalized to the present pandemic of a respiratory virus (SARS-COV-2) and it is recommended to use a facemask or respirator for reducing the spread of this respiratory virus.

## 1. Introduction:

In recent decades, humans have been threatened by a variety of viruses that lead to acute respiratory infections affecting human life and human societies, sometimes leading to death. There are several routes of respiratory virus transmission: contact, droplet, and aerosol. Respiratory viral infections cause a wide range of overlapping symptoms, known as acute respiratory illness (ARI) or, usually (more commonly) “the common cold” as a whole, which is chiefly mild but may cause severe illness and death (1). To protect themselves against such viruses, humans have offered a variety of strategies, from wearing facemasks and home quarantine to producing drugs and making the body resistant to such particles using appropriate vaccines. When specific vaccines or disinfection treatments are not available, the use of non-drug interventions, such as wearing respiratory personal protective equipment (RPPE), is important for protecting and reducing the occupational risk of health care workers (HCWs) against respiratory infections (2). 

Common types of PPE include surgical masks and respirators. It should be noted that surgical masks are loose and disposable and create a physical barrier between the wearers' mouth and nose and environmental contaminants (large respiratory droplets or sprays of blood and body fluids). They are not designed to filter out small airborne particles and have very different level of protection (3, 4). In this regard, the results of some studies have shown that daily use of surgical masks in all areas of the hospital is important in reducing swine flu infection, such measures at least prevent touching the mucous membranes of the nose and mouth with the fingers and such unconscious behaviors are less common (5, 6). These masks should be replaced as soon as they get wet or at least every four hours (3). In contrast, n95 respirators or their European equivalent, FFP2-3 prevent the penetration of at least 95% of aerosols less than 5 mm. But due to resistance to respiration and heat their long-term use is intolerable for HCWs (4). They should be worn for less than 8 hours during the day and should not be reused if they get wet (3). Besides, the equipment needs to fit completely on the face. It is very difficult to use this equipment in people with facial hair or beard and children, and is not recommended for the elderly, claustrophobic, and people with lung disease. Powered air-purifying respirators (PAPRs) have blowers that provide positive pressure airflow through the filter. They do not need to fully fit, and they protect the head and neck contiguously. One of its disadvantages is being the most expensive PPE. 

The world is currently suffering from a pandemic (7), caused by a virus now known as Severe Acute Respiratory Syndrome Coronavirus 2 (SARS-CoV-2) (named by the International Committee on Taxonomy of Viruses (ICTV)), Which has a phylogenetic similarity to SARS-CoV and its resulting disease is called COVID-19 (8, 9). Recommendations for the use of surgical masks during the present coronavirus disease pandemic include: people with suspected or confirmed respiratory infection COVID-19 symptoms; people in contact with HCWs or first-aid workers, and HCWs in contact with people with symptoms of respiratory infection (3). The present study aims to assess the effect of wearing a facemask on the reduction of incidence and prevention of infection with respiratory viruses such as SARS-CoV-2.

## 2. Methods:

This systematic review is reported based on the Preferred Reporting Items for Systematic Reviews and Meta-Analyses (PRISMA) guidelines (10).

**2.1. Search strategy**

 In this systematic review, Google Scholar, PubMed, Embase, Cochrane Library, Scopus, ProQuest, and Web of Science (WoS) were searched for articles published between 2009 and 2020. Boolean operators such as "AND" and "OR" were used to make different combinations for search (11). In addition, we searched using the following terms:

1--"facemask" [Mesh] OR "facemasks" [Mesh] OR "mask" [Mesh] OR “N95 Respirator” [Mesh] OR “Respirator, N95” [Mesh] OR “N95 Face Masks” [Mesh] OR “Face Mask, N95” [Mesh] OR “Mask, N95 Face” [Mesh] OR “N95 Face Mask” [Mesh] OR “N95 Masks” [Mesh] OR “Mask, N95” [Mesh] OR “N95 Mask” [Mesh] OR “N95 Filtering Facepiece Respirators” [Mesh] OR “N95 FFRs” [Mesh] OR “N95 FFR” [Mesh] 

2-"respirators"[Mesh] OR"Device, Respiratory Protective" [Mesh] OR "Protective Device, Respiratory" [Mesh] OR "Respirators, Industrial"[Mesh] OR "Respirators, Air-Purifying"[Mesh] 

3-"respiratory virus"[Mesh] OR " Severe Acute Respiratory Syndrome Virus" [Mesh] OR "SARS-Related Coronavirus" [Mesh] OR "SARS-CoV"[Mesh] "SARS Coronavirus"[Mesh] OR "SARS-Associated Coronavirus"[Mesh] OR "Coronavirus, SARS-Associated"[Mesh] OR "SARS Associated Coronavirus"[Mesh] 

4-#1 AND #2 AND #3

**2.2. Type of studies**

All studies published between 2009 and 2020, which were conducted to assess the effect of facemask on preventing the of spread respiratory viruses and reported the number or percentage of participants using a facemask and getting or not getting infected with a respiratory virus, were included in the study. Publications such as reviews, letters, comments, and case reports and studies that were conducted just for comparing different types of facemasks, or evaluated people with a tissue graft, or their sample size was not clear were excluded from the study. There were no language restrictions for using and entering articles in this study. If the language used in an article was not Persian or English, we asked a translator to translate the article.

**2.3. Types of participants**

The studies were selected if their participants were:

-people in contact with those infected with respiratory viruses

-people who were members of specific groups such as healthcare personnel, emergency department and general ward staff, public health workers, Hajj pilgrims

**2.4. Types of interventions**

The studies were reviewed if:

- participants used PPE

-measured the effect of facemask or PPE on preventing transmission of respiratory viruses 

**2.5. Type of outcome measure**

All studies measured the number and percentage of participants who used facemask, PPE, vaccine, and distance or did not use them and were infected after being in contact with a person infected with respiratory viruses. 

**2.6. Study selection**

The title and abstract of all studies retrieved during the electronic and manual follow-up search process were assessed based on the inclusion criteria. The full texts of relevant papers were examined based on the mentioned criteria.

**2.7. Quality assessment**

In this study, the quality of Cohort and Cross-Sectional studies was assessed according to the Newcastle-Ottawa scale (NOS) (12); and the quality of Controlled-Trial studies was assessed based on CONSORT. A maximum of ten stars was given to each study based on the NOS. A maximum of five stars was given to selection (including sample size, non-respondents, and ascertainment of the exposure). A maximum of two stars was given to comparability (including the study control for the most important factor). A maximum of three stars was given to outcome (including assessment of the outcome and statistical test). Studies of high-quality score nine or ten stars, studies with a score of seven or eight stars were considered to be of medium quality, and studies scoring less than six stars were considered to be of low quality (13). The CONSORT checklist was also used to report the standard clinical trial studies. This checklist contains 24 questions and a score of 0 or 1 is given to every question. If a study scored above 15, it was included in the study and thoe scoring 15 and below were excluded (14). The quality score for each article is shown in Table 1.

**2.8. Data extraction**

Two investigators independently searched for relevant scientific publications, carried out validity assessments, and resolved any disagreements by consulting a third investigator (15). Data were collected as follows:

1. Research information (reference, type of study, location, and sample size (Male, Female))

2. Characteristics of the participants (population, and age)

3. Intervention and comparison of the details (type of viruses, type of contact with an infected person, PPE type, facemask type)

4. Outcome measures (number of PPE types, number of facemask types, infected participants using facemask, distance, time of contact with person, time of using facemask, and vaccinated people)

**2.9. Statistical analysis**

We calculated the number and percentage of infected and non-infected people in all included studies and reported them in tables.

## 3. Results:

**Figure 1:**
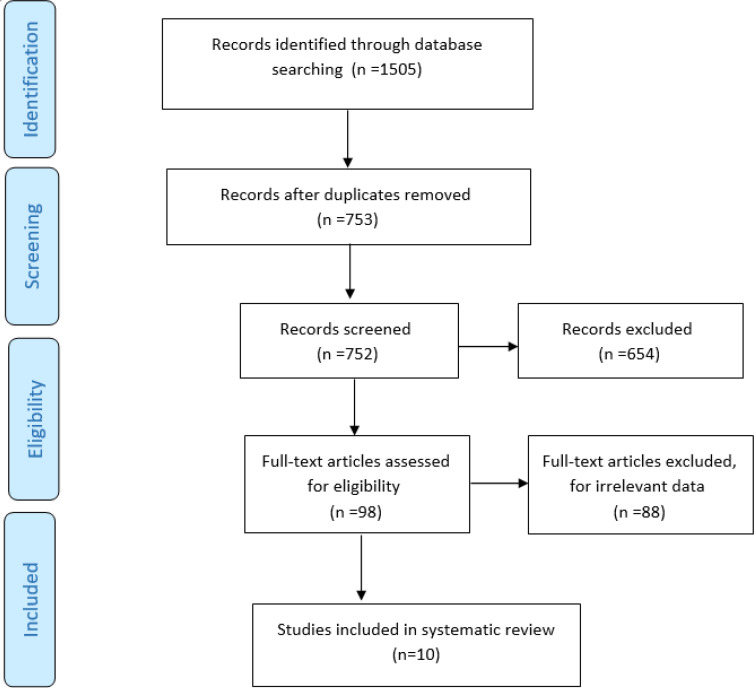
Flowchart of the review

The systematic search in the databases identified 1505 articles. After reviewing their titles and abstracts, 753 duplicate articles, 654 records with undesirable study types, and 88 irrelevant articles were removed. Finally, 10 articles (Sample Size=3065) were included in the systematic review. The flowchart of studies included in this review is shown in Figure 1. The characteristics of included studies are presented in Table 1 and their main findings are shown in other tables. The most frequent places where the studies were conducted were California (16, 17), Korea (18, 19), Saudi Arabia (20, 21), Thailand (22), Germany (23), Australia (24), Sydney (25), and New South Wales (25), respectively.

**3.1. Factors examined in the studies**

The factors presented in table one include the author’s name, study design, type of population, sample size, age, result, and quality score. Table two includes type of virus, type of contact with an infected person, PPE type, facemask type, and infected participants who used masks after contact with the patient. 

**3.2. Type of virus, contact, PPE, facemask, and the number of infected people who used a facemask**

**Type of Virus**

The types of virus assessed in this systematic review included SARS-CoV-2 (16), MERS (18), MERS-CoV (19, 20, 22), Rhinovirus (21, 24, 25), Influenza A viruses (H1N1) (17, 21, 23-25), Influenza B viruses (21, 23-25), Parainfluenza 1,2 and 3 viruses (21, 24), Enteroviruses (21), Adenoviruses (24, 25), Human metapneumoviruses (24, 25), Respiratory syncytial viruses A or B (25), Coronaviruses (24, 25), Picornaviruses (25), and Enteroviruses (25).

**3.3. Type of Contact**

The type of contact with an infected person was assessed in nine articles and varied in different studies. Generally, the contact was between health care workers and infected persons (16, 17, 20, 22, 24), emergency department and general ward (18), public health workers (19), Hajj pilgrims (21), and household contact (23, 25), contact with aerosol, and skin-to-skin contact.

**3.4. PPE Type**

The PPE type was mentioned in all assessed studies. PPE types in the studies included gloves (16-20, 22), kind/various types of facemask (16-25), N95 respirator (17, 18, 20), gown (17, 20, 22), face shield, eye protection (20, 22), and cap (22).

**3.5. Type of Facemask**

The type of facemask was mentioned in nine studies and the number of participants who wore each type of facemask was mentioned in seven studies. Surgical masks (18, 19, 21, 23, 25) were used by 18.8%, N95 respirators (17-20, 22, 24) by 34%, Medical masks (20) by 16.1%, and P2 masks (25) by 3% of participants.

**3.6. The number of infected people who used a facemask or N95 respirator**

Generally, 3.2% of participants who wore a facemask became infected with the respiratory virus through contact with an infected person. In studies that all participants wore surgical masks (18, 19, 21, 23, 25), 0.3% of them became infected. In studies that all participants wore N95 respirators (17-20, 22, 24), 1.4% became infected. 0.2% of participants who wore N95 respirators and surgical masks became infected (18, 19). 1.7% of participants who wore N95 respirators and medical masks became infected (20). 0.3% of participants who wore N95 respirators and other facemasks became infected (17) and 0.5% of participants who wore P2 masks and surgical masks became infected (25). Also, some studies did not mention the effect of each facemask on preventing virus spread and the percentage of using each type of facemask so it was not possible to draw a precise conclusion about the quality and effectiveness of each type of facemask based on the mentioned statistics.

**3.7. Distance and time of contact**

Distance between people and the duration of contact with an infected person was mentioned in five studies (16, 19-22). We could not assess the effect of distance and duration of contact on preventing the spread of virus because these studies did not mention the count of the infected participants based on distance and duration of contact.

**3.8. Duration of using a facemask and vaccinated**

The duration of facemask use and the number of vaccinated people were evaluated in five studies (17, 20, 23-25). However, these studies did not mention the effect of vaccination and duration of facemask use on prevention of virus infection. 

## 4. Discussion:

The results of the present study showed that using facemask or respirators has a preventive effect on the spread of respiratory viruses including SARS-CoV-2, MERS-CoV, Rhinovirus, Influenza A virus (H1N1), Influenza B viruses, Parainfluenza 1,2 and 3 viruses, Enteroviruses, Adenoviruses, Human metapneumoviruses, Respiratory syncytial viruses A/B, Coronaviruses, Picornaviruses, and Enteroviruses. Moreover, the percentage of transmission of respiratory viruses in people who wore any type of facemask or N95, respectively, was 0.3% in the surgical mask group, 1.4% in the N95 respirators group, 0.2% in N95 respirators and surgical mask group, 1.7% in N95 respirators and medical mask group, 0.3% in N95 respirators and another facemask group, and 0.5% in P2 masks and surgical mask group.

In COVID-19 disease pandemic, due to the lack of access to appropriate antiviral medications and the lack of a suitable vaccine, infection control was the most important way to control it (26). Therefore, many countries performed non-drug interventions and early control strategies that included the use of facemasks and double key actions such as hand hygiene and temporary closure of schools and offices as recommended by the international scientific communities (25). SARS-CoV-2 transfer is mainly through direct transfer of droplets by sneezing and coughing and contact; also, airborne transfer is possible via aerosol-generating procedures (AGPs) (24, 27, 28). Moreover, it has been shown that large droplets could accelerate the transfer of fomite and suspended in the air (28). Besides, these large droplets can move up to two meters or eight meters (28). In this regard, Liu et al. showed that a high concentration of virus is in the air sample of the patient’s toilet and in the environment in which HCWs their PPE (29). Hence, virus-infected particles are more likely to be suspended in the air in places where the airflow is turbulent and patients with or without symptoms or in the pre-symptom phase of COVID-19 have many referrals (30). And health care workers are more likely to become infected (46).

 In this regard, the rate of Influenza virus transmission between HCWs in the H1N1 pandemic were 14%, yet 67% of them did not have any signs (31). Besides, most positive cases were between outpatient and Ancillary HCWs and HCWs who used surgical masks or N95 respirators were seronegative (31). This indicates that they’d not use PPE such as facemasks (31). Moreover, Ki et al. showed that emergency department staff had more exposure to respiratory infections MERS disease compared to the internal department staff (In terms of time, the medical staff of the emergency department spent more time with the index patient than those working in the internal department, eight hours versus one hour). However, the rate of being stricken with disease was 3% vs 16.7%, respectively, because 93% of emergency department staff used surgical masks and 95% of them used hand wash but only 8% of the general department staff used surgical masks (32). 

It should be noted that in studies, the proportion of infected HCWs among the confirmed cases of Coronavirus disease-2019 is 10% in Italy, 20% in Spain, and 40% in the United States (33). Infected HCWs can be a source of infection for other HCWs and patients (4). Therefore, the protection of HCWs and the provision of PPE are of particular importance to maintaining ongoing care and the function of the health care system. On another hand, Liu et al. showed that the rate of RNA virus was reduced by performing a disinfection procedure in the PPE room (29). This emphasizes the effect of environmental disinfection in reduction of virus transmission (29). 

Therefore, in addition to providing PPE for HCWs, health organizations should emphasize continuous training on donning and doffing for effective protection (34, 35). Study results showed that the infection control team of the hospital can significantly reduce the transmission of the disease between HCWs through early diagnosis and identifying index cases among patients and explaining preventive actions to them, giving early leave to sick staff, or at least giving them some recommendations such as maintaining social distance with other staff, hand hygiene observation, and wearing facemask during a disease outbreak, and monitoring the adherence to infection control methods (26). In addition to HCWs’ awareness, climate condition is another factor that obstructs the correct use of respiratory protection equipment between HCWs (36). 

Studies state that in a household setting, due to the increased cumulative exposure time of index patients with family members, the possibility of transmitting viral respiratory infections is higher (37, 38). In this regard, results of studies showed that if family members perform non-drug interventions (such as wearing facemask and maintaining hand hygiene for the prevention of home flu), during 36 hours after onset of symptoms in the index case, the incidence of influenza will be significantly lower in family members compared to control group. One of the possible reasons is that the virus shedding often occurs during the first 36 hours so if the preventive interventions start sooner, the probability of home transmission of disease will be lower. Moreover, this study showed that the permanent use of facemask is tolerable and acceptable for both adults and children. In this regard, another study on the effect of non-drug interventions (wearing facemask and hand hygiene) in home environments in Bangkok, Thailand failed to see any effect. Of course, it should be mentioned that index patients who were infected with Influenza (children) slept with their parents without wearing facemask throughout the night, which may block any effect of interventional protection during the day (39).

In a random retrospective cluster clinical trial study, MacIntyre et al. revealed that less than 50% of participants had the necessary compliance for wearing a facemask and in this group had a significantly lower risk of Influenza-like illness due to infection (25). Results of another study in a social setting such as a student dormitory showed that influenza-like illness was significantly less in the group that used a facemask, and the group that both used a facemask and maintained hand hygiene compared to the control group. They suggested that adding hand disinfectant does not increase the protective efficacy of wearing a facemask or at least does not increase it significantly (40). Results of these studies show that if index patients and people around them do non-drug interventions such as wearing a facemask and washing their hands in domestic or social settings during the first 36 hours, these can significantly reduce the incidence of respiratory infection in people around them. 

One of the important religious rituals is Hajj, in which large numbers of people from different countries congregate in a particular region. Thus, it naturally increases the risk of transmitting respiratory viral infections. Results of studies show that use of facemasks by people with the influenza-like illness and those around them (those who were in a tent together) provided more protection against the Influenza-like illness compared to those in the control group, who did not wear protective equipment, and the percentage of infection in each group was 31% vs. 53%, respectively. A significant point in this report was the presence of a positive correlation between duration of facemask use and protection against influenza-like illness, so the incidence of disease in pilgrims who wore facemask more than eight hours a day compared to those who wore a mask for less than eight hours a day was 3% vs 43% (21). 

Currently, due to the intensity of the COVID-19 pandemic, wearing a facemask is common everywhere, including hospitals and communities (3). In this regard, the results of studies indicate that wearing a facemask is more effective in controlling the spread of infection, especially from asymptomatic carriers. The inward efficacy surpasses outward efficacy and the public’s use of facemasks plays a greater role in controlling the source of infection. Of course, wearing a facemask is recommended as a method of infection control and primary prevention for healthy persons (26, 41). Lai et al showed that the use of facemask by HCWs is logical and the use of facemasks, even low-quality cloth masks, by the public should be implemented in the community, immediately. If people can use medical masks, without medical staff running out of PPE reserves, it will be more effective in the prevention of disease transmission. The use of cloth masks by patients or infectious individuals without clinical symptoms prevents infection transmission in the community. Of course, the filtration effects of cloth masks are generally lower than surgical masks. However, if they are well designed (multi-layered cloth masks made of water-resistant fibers with high yarn density and delicate cloth) and used properly (so that the whole face fits in it) they may provide reasonable protection. It has also been shown that these masks can decrease virus exposure, although their ability is much less than medical masks. Therefore, HCWs should not use this type of mask, since the results of studies show that the risk of infection among HCWs who use cloth masks is higher than those who use the medical mask or control group (42). Thus, CDC expressed that at the time of COVID-19 pandemic and due to the limited resources of medical masks, the public could use hand-made cloth masks to optimize facemask stocks. Also, they should be advised to wash their hands daily with hot water and soap, and other appropriate methods. Besides, the public should be educated about their use (27).

Therefore, PPE should be selected for special settings and they should be used logically (4). It was thought that N95 respirators have more efficacy in filtering very small particles than surgical masks. However, the results of studies show that in non-infectious health care settings, surgical masks do not have lower protective efficacy than N95 respirators in HCWs (43-46). However, results of one cluster clinical trial showed that the rate of respiratory infections in HCWs who always use medical mask was double compared to HCWs who always used N95 respirators, and the highest rate of infection was among HCWs who had close contact with patients such as radiologists, followed by nurses. Nevertheless, that study did not have enough power for the results to be generalized, since the rate of observed infection was much lower than expected (24). 

**Table 1:** Overview of all the studies included in this systematic review

Reference	Study design	Location	Population	Sample size (N, %)	Age (year)	Result	Quality score
Amy Heinzerling (2020)(16)	Cohort	California	Healthcare Personnel	T:43 M: 16% F: 84%	27 – 60	Reducing the risk of SARS-CoV-2 transmission using Patient source control (e.g., a patient wearing a facemask or connected to a closed-system ventilator during HCP exposures)	7*
Hyun Kyun Ki (2019)(32)	Case–Cohort	Korea	Emergency department and general ward	T: 446 M: 46.9% F: 53.1%	20 - 78	Great reduction in nosocomial transmission of MERS by routine infection-prevention policies such as wearing a surgical mask and hand hygiene	9*
Boyeong Ryu (2018)(19)	cross-sectional	Korea	Public health workers	T: 34 M: 58.8% F: 41.1%	34 – 56.7	Properly use of PPE lead to a lack of evidence of MERS on Public Health Provider	7*
Basem M. Alraddadi (2016)(20)	Cohort	Saudi Arabia	Healthcare Personnel	T: 250 M: 64.4% F: 35.6%	18 - 66	- more protective against MERS CoV infection while in close contact with an infected patient by N95 respirators - highlight the possible role of short-range aerosol transmission of MERS-CoV in healthcare settings	7*
Surasak Wiboonchutikul (2016)(47)	Cross-sectional	Thailand	Healthcare workers	T:38 M: 21.1% F: 78.9%	38.6	Healthcare workerscan be protected via strict infection control precaution	7*
Osamah Barasheed (2014)(21)	Controlled trial	Saudi Arabia	Australian Hajj Pilgrims	T: 164 M: 43.3% F: 56.7%	19-80	The positive association between the duration of facemask use and protection against ILI	20**
Thorsten Suess (2012)(38)	Controlled trial	Germany	Households	T: 302 M: 51.6% F: 48.3%	4-43	Interruption of influenza transmission within households by using a facemask	22**
Jenifer L. Jaeger (2011)(31)	Cohort	California	Healthcare Personnel	T: 63 M: 23.8% F: 76.2%	19–74	A significant association between using facemask or N95 respirator and seronegative and asymptomatic respiratory disease	8*
Chandini Raina MacIntyre (2011)(24)	Controlled trial	Australia	Health care workers	T: 1441 M: 9.9% F: 90.1%	≥ 18	- Approximately double rates of respiratory tract infection in the medical mask group compared to the N95 group among healthcare workers - Signiﬁcant protection against CRI using N95 non-ﬁt tested arm	21**
Chandini Raina MacIntyre (2009)(25)	Controlled trial	Sydney, New South Wales, Australia	Households	T: 284	≥ 0	The important role of using a facemask in preventing transmission	20**

T: Total, M: Male, F: Female, PPE: Personal Protective Equipment, ILI: Influenza-like illness; HCP: Healthcare personnel ; CRI: clinical respiratory illness.

*By Newcastle-Ottawa scale (NOS)

**By Consolidated Standards of Reporting Trials (CONSORT)

**Table 2:** Type of virus, contact, personal protective equipment (PPE), and facemask, and the number of people infected despite using a facemask in studied articles

Reference	Type of virus	Type of contact with an infected person	PPE type (%) P_value	Facemask type (%)	Infected participants using a facemask and in contact with the patient (N) (%)	
Amy Heinzerling (2020)(16)	SARS-CoV-2	Taking vital signs	Gloves^* ^(64.9%)	NR^**^	Yes:8.1%	No:91.9%
		Taking a medical history				
		Performing a physical exam	Facemask (8.1%)			
		Providing medication				
		Bathing or cleaning patient				
		Lifting or positioning patient				
		Emptying bedpan				
		Changing linens				
		Cleaning patient room				
		Peripheral line insertion				
		Central line insertion				
		Drawing arterial blood gas				
		Drawing blood				
		Manipulation of oxygen mask or tubing				
		Manipulation of ventilator or tubing				
		In-room while high-flow oxygen being delivered				
		Collecting respiratory specimen				
Hyun Kyun Ki (2019)(32)	MERS	Touch of patient	Mask or respirator (52.9%) P_value<0.05	Surgical mask(48.4%) P_value<0.001	Yes:0.8%	No: 99.1%
		Touch of bed or equipment	Gloves (0.8%) P_value=0.624	N95 respirator (4.4%)		
Boyeong Ryu (2018)(19)	MERS-CoV	Patient transportation	Facemask (100%)	N95 respirator in participants (100%)	Yes:2.9%	No: 97.1%
		Patient counseling				
		Ambulance disinfection				
		Specimen transportation				
		Respiratory specimen collection	Gloves	Surgical mask in symptomatic patients		
		Taking vital signs				
		Discarding exposed goods				
		Other				
Basem M. Alraddadi (2016)(20)	MERS-CoV	Direct contact with patient	Gloves (87.2%)	Medical mask P_value>0.05	Yes:6.4%	No: 93.6%
			Gown (87.2%) P_value=0.81			
		Aerosol-generating procedures	Eye protection P_value>0.05	N95 respirator P_value>0.05		
			Facemask or respirator P_value>0.05			
Surasak Wiboonchutikul (2016)(47)	MERS‐CoV	Touching the patient	Gown (%100)	N95 respirator (100%)	Yes:0%	No:100%
		Touching the patient’s equipment	Gloves (%100)			
		Examining clinical specimens	Eye protection (%100)			
		Obtaining clinical specimens	Cap (%100)			
		Cleaning the patient’s room	Facemask (%100)			
Osamah Barasheed (2014)(21)	Rhinovirus	Usual contact between people in Hajj	Facemask (45.7%) P_value>0.05	Surgical mask (45.7%) P_value>0.05	Yes:11.1%	No:88.8%
	Influenza A (H1N1)					
	Influenza B Dual infection (rhino & influenza A)					
	Parainfluenza 3					
	Enterovirus					
Thorsten Suess (2012)(38)	Influenza A (H1N1)	Household contacts	Hygiene and facemask (31.4%) P_value=0.2	Surgical mask (62.9%) P_value<0.05	Yes:8.6%	No:91.3%
	Influenza B		Facemask (62.9%) 0.3			
Jenifer L. Jaeger (2011)(31)	Influenza A (H1N1)	Encounters	Gloves (71.4%) P_value>0.05	Mask P_value>0.05	Yes:14%	No:86%
		Exposure to respiratory secretions	Gown P_value>0.05			
		Exposure before institution of at least Droplet Precautions	Facemask or N95 respirator (31.7%) P_value>0.05	N95 respirators P_value>0.05		
		Skin-to-skin contacts	Total PPE use (73%) P_value>0.05			
Chandini Raina MacIntyre (2011)(24)	Adenoviruses	NR	Facemask (100%) P_value=0.19	N95 ﬁt (32%) P_value=0.19	Yes:1.8%	No: 98.2%
	Human metapneumovirus					
				N95 non-ﬁt (33.9%) P_value=0.03		
	Coronavirus 229E⁄NL63					
				Medical mask (34.1%) P_value=0.67		
	Parainﬂuenza viruses 1, 2 or 3					
	Inﬂuenza viruses A (H1N1) or B					
	Respiratory syncytial virus A or B					
	Rhinovirus A⁄B					
	Coronavirus					
Chandini Raina MacIntyre (2009) (25)	Inﬂuenza A (H1N1)	Household contacts	Facemask (65.4%) P_value=0.19	Surgical mask (33%) P_value=0.32	Yes:8%	No:92%
	Inﬂuenza B					
	Adenoviruses		Daily hand wash P_value=0.21	P2 mask (32.4%) P_value=0.12		
	Respiratory syncytial virus		Using soap for washing hand P_value=0.87			
	Parainﬂuenza viruses 1,2 and 3					
	Human metapneumovirus					
	Coronavirus OC43					
	Picornaviruses					
	Rhinoviruses					
	Enteroviruses					
	Uncharacterized no					
	Sequenced picornaviruses					

*These percentages were reported from 37 health care workers who were tested for SARS-CoV-2 and participated in interviews in this study.

** NR: not reported

**Table3:** Quality assessments of cotrolled clinical trials ,based on CONSORT

Item	Osamah Barasheed (28)	Thorsten Suess (45)	Chandini Raina MacIntyre (31)	Chandini Raina MacIntyre (32)
1.Identification as a randomized trial in the title structured summary of trial design, methods, results, and conclusions (for specific guidance see CONSORT for abstracts)	1	1	1	1
2. Scientific background and explanation of rationale, specific objectives, or hypotheses	1	1	1	1
3. Description of trial design (such as parallel, factorial) including allocation ratio Important changes to methods after trial commencement (such as eligibility criteria), with reasons	1	1	1	1
4. Eligibility criteria for participants Settings and locations where the data were collected	1	1	1	1
5. The interventions for each group with sufficient details to allow replication, including how and when they were actually administered	1	1	1	1
6. Completely defined pre-specified primary and secondary outcome measures, including how and when they were assessed Any changes to trial outcomes after the trial commenced, with reasons	1	1	1	1
7. How sample size was determined when applicable, explanation of any interim analyses and stopping guidelines	1	1	1	1
8. Method used to generate the random allocation sequence, type of randomisation, details of any restriction (such as blocking and block size)	1	1	1	1
9. Mechanism used to implement the random allocation sequence (such as sequentially numbered containers), describing any steps taken to conceal the sequence until interventions were assigned	1	1	1	1
10. Who generated the random allocation sequence, who enrolled participants, and who assigned participants to interventions	1	1	1	1
11. If done, who was blinded after assignment to interventions (for example, participants, care providers, those assessing outcomes) and how If relevant, description of the similarity of interventions	0	1	0	0
12. Statistical methods used to compare groups for primary and secondary outcomes Methods for additional analyses, such as subgroup analyses and adjusted analyses	1	1	1	1
13. For each group, the numbers of participants who were randomly assigned, received intended treatment, and were analysed for the primary outcome For each group, losses and exclusions after randomisation, together with reasons	1	1	1	1
14. Dates defining the periods of recruitment and follow-up Why the trial ended or was stopped	1	1	1	1
15. A table showing baseline demographic and clinical characteristics for each group	1	1	1	1
16. For each group, number of participants (denominator) included in each analysis and whether the analysis was by original assigned groups	1	1	1	1
17. For each primary and secondary outcome, results for each group, and the estimated effect size and its precision (such as 95% confidence interval) For binary outcomes, presentation of both absolute and relative effect sizes is recommended	0	0	0	0
18. Results of any other analyses performed, including subgroup analyses and adjusted analyses, distinguishing pre-specified from exploratory	0	1	0	1
19. All-important harms or unintended effects in each group (for specific guidance see CONSORT for harms)	1	1	1	0
20. Trial limitations, addressing sources of potential bias, imprecision, and, if relevant, multiplicity of analyses	1	0	1	1
21. Generalisability (external validity, applicability) of the trial findings	1	1	1	1
22. Interpretation consistent with results, balancing benefits and harms, and considering other relevant evidence	1	1	1	1
23. Registration number and name of trial registry	0	0	0	0
24. Where the full trial protocol can be accessed, if available	0	0	0	0
25. Sources of funding and other support (such as supply of drugs), role of funders	1	1	1	1
Total Score	20	22	21	20

**Table 4:** Quality assessments of Cohort studies (using NEWCASTLE - OTTAWA)

Study	Year	Study type	Selection	Comparability	Outcome
Amy Heinzerling (17)	2012	Cohort	****	-	***
Basem M. Alraddadi (21)	2020	Cohort	***	*	***
Jenifer L. Jaeger (32)	2012	Cohort	***	*	***
Hyun Kyun Ki (33)	2011	Cohort	*****	*	***
Boyeong Ryu (20)	2010	Cross-Sectional	***	**	**
SurasakWiboonchutikul (48)	2016	Cross-Sectional	***	**	**

## 5. Limitations

The limitations of this study included lack of access to full-text of some studies, lack of data on the effect of facemasks on prevention of each type of respiratory viruses in articles that had studied different viruses, the lack of data on the effect of each type of facemask on reducing the transmission of respiratory viruses and count or percentage of participants who used a facemask. Moreover, some studies conducted on recent viruses (SARS-Cov-2) were not published in English, which is another limitation of the study.

## 6. Conclusion:

This systematic review showed that using facemasks or respirators aided in preventing the spread of respiratory viruses. The result of the present systematic review showed that using facemasks could prevent the spread of virus. We recommend conducting more studies on the effect of each type of facemask and respirator, individually, and on the prevention of the spread of different viruses. Moreover, we suggest assessing the effect of simultaneous use of masks, duration of using a facemask, and distance between healthy people and the person infected with respiratory viruses.

## 7. Declaration:

### 7.1. Acknowledgments

 The authors acknowledge Alborz University of Medical Sciences.

### 7.2. Authors’ contribution

FA and NSH fulfilled an advisory role. ZA carried out the literature survey. NSH edited this manuscript in English. MD commented on the manuscript. All authors passed the criteria for authorship contribution based on recommendations of the International Committee of Medical Journal Editors.

### 7.3. Funding information

This study did not receive any funding support.

### 7.4. Conflict of interest

The authors state no conflicts of interest.
